# Polymorphic Structures of Alzheimer's β-Amyloid Globulomers

**DOI:** 10.1371/journal.pone.0020575

**Published:** 2011-06-07

**Authors:** Xiang Yu, Jie Zheng

**Affiliations:** Department of Chemical and Biomolecular Engineering, The University of Akron, Akron, Ohio, United States of America; University of Dayton, United States of America

## Abstract

**Background:**

Misfolding and self-assembly of Amyloid-β (Aβ) peptides into amyloid fibrils is pathologically linked to the development of Alzheimer's disease. Polymorphic Aβ structures derived from monomers to intermediate oligomers, protofilaments, and mature fibrils have been often observed in solution. Some aggregates are on-pathway species to amyloid fibrils, while the others are off-pathway species that do not evolve into amyloid fibrils. Both on-pathway and off-pathway species could be biologically relevant species. But, the lack of atomic-level structural information for these Aβ species leads to the difficulty in the understanding of their biological roles in amyloid toxicity and amyloid formation.

**Methods and Findings:**

Here, we model a series of molecular structures of Aβ globulomers assembled by monomer and dimer building blocks using our peptide-packing program and explicit-solvent molecular dynamics (MD) simulations. Structural and energetic analysis shows that although Aβ globulomers could adopt different energetically favorable but structurally heterogeneous conformations in a rugged energy landscape, they are still preferentially organized by dynamic dimeric subunits with a hydrophobic core formed by the C-terminal residues independence of initial peptide packing and organization. Such structural organizations offer high structural stability by maximizing peptide-peptide association and optimizing peptide-water solvation. Moreover, curved surface, compact size, and less populated β-structure in Aβ globulomers make them difficult to convert into other high-order Aβ aggregates and fibrils with dominant β-structure, suggesting that they are likely to be off-pathway species to amyloid fibrils. These Aβ globulomers are compatible with experimental data in overall size, subunit organization, and molecular weight from AFM images and H/D amide exchange NMR.

**Conclusions:**

Our computationally modeled Aβ globulomers provide useful insights into structure, dynamics, and polymorphic nature of Aβ globulomers which are completely different from Aβ fibrils, suggesting that these globulomers are likely off-pathway species and explaining the independence of the aggregation kinetics between Aβ globulomers and fibrils.

## Introduction

Alzheimer's disease (AD) is a progressive and fatal neurodegenerative disease characterized by extracellular deposition of β-amyloid (Aβ) as senile plaques and intracellular accumulation of aggregated neurofibrillary tau tangles [1] in human brain. The primary components of the extracellular senile plaques are Aβ_1–40_ and Aβ_1–42_ peptides, which are produced by cleaving a transmembrane amyloid precursor protein (APP) at Asp672 in the extracellular domain by β-secretase and at Ala713 within the intracellular domain by γ-secretase [2]. Aβ_1–40_ is a more abundant species, whereas Aβ_1–42_ is more neurontoxic species. Both Aβ peptides have an extracellular hydrophilic N-terminus (residues 1–28) and a membrane-inserted C-terminus region (residues 29–40 or 29–42) [3]. Production of Aβ is a normal process, but overexpression of Aβ appears to cause the early onset of AD [4].

Accumulating evidence has shown that soluble Aβ oligomers are more neurotoxic than Aβ fibrils [5]. For example, a number of *in vivo* studies [6], [7], [8] have reported that synapse loss was well correlated with the level of soluble Aβ oligomers, but not monomeric Aβ, insoluble fibrils, or APP levels. Studies of transgenic mouse models also demonstrated that significant neuronal injury occurred before the appearance of amyloid plaques [9], [10]. Aβ oligomers can be classified by sizes from dimer, Aβ-derived diffusible ligands (ADDL), globulomers, and annular or pore-like aggregates [11], [12], [13], [14], [15]. Although the concrete toxic mechanism of soluble Aβ oligomers still remain elusive, it is generally accepted that the spontaneous aggregation of Aβ oligomers has deleterious effects on the neuron cell membrane with severe consequences for perturbing ionic homeostasis, triggering oxidative injury, and altering signaling pathways [16], [17].

It is well known that both amyloid oligomers and fibrils display a wide variety of structural morphologies, resulted from peptide packings and conformations, local cross-section architecture, and overall symmetry [18], [19]. But, there exists a gap between high-resolution structures for Aβ fibrils determined by X-ray and solid-state NMR [20], [21] and low-to-intermediate resolution structures for Aβ oligomers of varied morphologies by AFM and EM [22], [23]. Several NMR-based Aβ_1–40_ and Aβ_1–42_ models have been determined for Aβ fibrils [18], [20], [21]. But, little structural information is currently known about amyloid oligomers, because of their small sizes, transit short-lived time, heterogeneous structures, and notorious structural sensitivity depending on experimental conditions and sample preparation methods [24], [25], [26]. Thus, obtaining structural information about Aβ oligomers at the atomic-level is the first and important step to understand the structure-toxicity relationship of AD and other amyloidogenic diseases.

Specifically, Aβ-derived diffusible ligands (ADDL) and Aβ globulomers that appear at early stages of Aβ aggregation have been found to be highly toxic to neurons by altering synaptic activity [12], [27]. Barghorn et al. [15] have reported that Aβ globulomers primarily exist as 12-mers after short incubation time. They are very stable and inert to assist amyloid fibril formation upon long-time incubation, implying that the formation of Aβ globulomers and fibrils undergoes different kinetic pathways (11, 15, 26). Yu et al. [28] recently proposed a low-resolution structural model for Aβ_1–42_ globulomers consisting of dimer units, but no detailed atomic structure of Aβ_1–42_ globulomer was provided.

**Figure 1:**
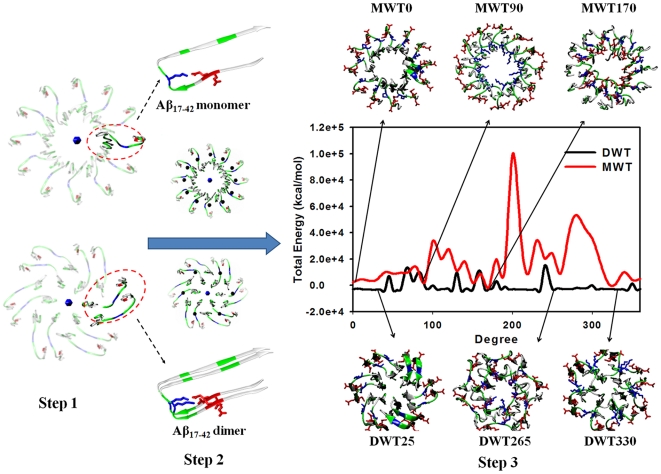
A three-step assembly procedure for constructing Aβ_17–42_ globulomers by using monomer or dimer building blocks. Step 1: Aβ monomer/dimer aligns parallel to the z axis (i.e. core axis) and then is rotated and replicated to form an annular structure. Step 2: each building block (i.e. monomer or dimer) is self-rotated along its β-strand axis at the center of mass by 5° interval from 0° to 360° to generate 72 candidates. Step 3: each candidate is energy minimized using GBSW implicit solvent model to obtain preliminary energy profiles of monomer-based globulomers (*red*) and dimer-based globulomers (*black*). Six lowest-energy globulomers with different peptide packings are preselected as initial conformations for subsequent explicit-solvent MD simulations to examine their structural stability.

In this work, we modeled a series of Aβ globulomers by systematically searching different packing possibilities of 12 NMR-derived β-strand-loop-β-strand Aβ peptides using an in-house peptide-packing program and explicit-solvent molecular dynamics (MD) simulations, which have been proved to be effective in determining various Aβ oligomers of annulars [29], triangulars [30], and micelles [31]. Aβ globulomers were built by parallel aligning 12 Aβ monomers or 6 Aβ dimers into annular structures, and classified into monomer-based globulomers with 12-fold symmetry and dimer-based globulomers with 6-fold symmetry (Figure 1). Three monomer-based models and three dimmer-based models with the lowest packing energies were coarsely selected from 72 candidates using our peptide-packing program with the generalized born of a simple switching function (GBSW) implicit-solvent model. Six globulomer models were then subject to explicit-solvent MD simulations to examine the effect of structural symmetry and building block on the structural and energetic aspects of Aβ globulomers. Simulation results showed that although Aβ globulomers tended to develop into different rigid and heterogeneous structures formed by dimer subunits, they all contained a hydrophobic core by C-terminal β-strands shielding from the solvent. These globulomers substantially differed from the high-order Aβ aggregates in β-structure population, but consistent with experimental observations in several aspects including size, subunit organization, and residue solvation. Knowledge from detailed atomic structures of Aβ globulomers in this work suggests that Aβ globulomers are off-pathway species to amyloid fibrils and other oligomers, suggesting the independence of the aggregation kinetics between Aβ fibrils and Aβ globulomers.

## Materials and Methods

### Model construction

Although there is no atomic structure of Aβ globulomers available to date, experimental characterization of Aβ_1–42_ globulomers by Barghorn et al. [11], [15] and Yu et al. [28] has shown that (i) Aβ globulomers mainly consists of 12 Aβ monomers; (ii) Aβ globulomers display a circular shape with heights of 4–6 nm by AFM images; and (iii) Aβ globulomers form a hydrophobic inner core via hydrophobic C-terminal β-strands, while hydrophilic N-terminal β-strands are exposed to bulk solvent. Based on this structural information, we used Aβ monomer or Aβ dimer as building blocks to construct circular-like globulomers consisting of 12 peptides because monomer and dimer are the most common building units in biomacromolecules [32], [33]. Since 1–16 residues of Aβ_1–42_ peptide are disordered and not atomic structure is available to date, we only used residues 17–42 of Aβ to generate Aβ globulomers. Aβ_17–42_ monomer was generated by averaging Aβ_17–42_ NMR-derived structures (PDB code 2BEG) [20], consisting of two antiparallel β-strands (residues 17–25 and 31–42) linked by a U-turn region (Ser_26_-Asn_27_-Lys_28_-Gly_29_-Ala_30_). Aβ_17–42_ dimer building block was generated by stacking two Aβ_17–42_ monomers on the top of each other in an in-register manner, with an initial peptide-peptide separation distance of ∼4.7 Å (Figure 1).

Figure 1 shows a two-step procedure to build monomer-based Aβ globulomers. First, single Aβ_17–42_ monomer was aligned to the *z* axis with an initial radius of 10 Å from the origin of the Cartesian coordinate and was replicated and rotated along the *z* axis at every 30° to form an annular-like structure, as described in previous works [29], [34]. Then, each of 12 monomers was simultaneously rotated along its own β-strand axis at the center of mass at every 5° to generate 72 12-fold globulomers, which present significant structural differences in the geometry of the cross-section and the contact interface of sidechains. Finally, each candidate was subject to 1000 steps of energy minimization with the generalized born of a simple switching function (GBSW) implicit solvent model [35]. Three lowest-energy globulomers with distinct morphology (Figure 1) were selected and subjected to explicit-solvent MD simulations for examining their structural and energetic aspects at the early stage of the aggregation process. Following the same procedure, we used Aβ dimer, instead of Aβ monomer, as building units to generate dimer-based globulomers with varied sizes and shapes. It should be noted that dimer building block was first replicated and rotated at every 60° to form a 6-fold annular structure, followed by simultaneous rotation of every dimer unit by 5° to generate 72 6-fold dimer-based globulomers. All initial structures shared similar size, ring-like shape, and the same number of peptides.

### Explicit-solvent MD simulation

All MD simulations were performed by the NAMD program [36] using the CHARMM27 force filed, including dihedral cross-term corrections (CMAP) [37] for peptides and modified TIP3P water models. Counterions of NaCl were added to neutralize the systems. Simulations were performed using an NPT ensemble under periodic boundary conditions. Constant pressure (1 atm) and temperature (330 K) were maintained by an isotropic Langevin barostat and a Langevin thermostat. The simulation temperature of 330 K was slightly higher than room temperature and thus may aid in avoiding local energy traps and allow us to probe the stabilities and dynamics of Aβ globulomers more quickly in the limited simulation time. The long-range electrostatic interactions were treated by the Particle Mesh Ewald (PME) method using a real space cutoff of 12 Å and a grid size of ∼1 Å in all directions, and a fifth order β-spline was used for the interpolation. The short-range Van der Waals (VDW) interactions were smoothly switched off between 10 and 12 Å. All covalent bonds involving hydrogen were constrained with the SHAKE algorithm. The velocity verlet integrator with a time step of 2 fs was used to solve Newton's equations of motion. Nonbonded and image lists were updated every 20 integration steps. Using all 6 models and total 3000 conformers (500 conformers from each model) extracted from the last 10-ns MD simulations, an in-house Monte Carlo (MC) program was used to estimate the overall populations. Details of the simulations were summarized in Table 1.

**Table 1:** Structural details of different Aβ globulomer systems.

Models	Entanglement	RMSD(Å)/Rg (Å)	Energy (kcal/mol)	Population (%)
MWT0	No	7.7/18.8	−3860.7±30.4	27.5
MWT90	Yes	12.0/19.9	−3738.1±29.4	11.7
MWT170	No	14.3/21.8	−3674.2±25.8	3.3
MMT0	No	11.5/19.7	−3030.8±37.9	/
DWT25	No	7.0/18.8	−3780.9±26.8	18.5
DWT265	Yes	7.5/19.1	−3735.0±24.7	11.2
DWT330	Yes	7.1/18.6	−3863.3±22.0	27.8
DMT25	No	7.5/18.8	−2986.5±31.4	/

## Results

For clarity and convenience, a globulomer system is notated by the following sequences: the type of building block (i.e. single letter M for monomer building block and D for dimer building block)+the type of amino acid sequence (i.e. double letter WT for wild-type and MT for mutant)+the self-rotation degree of each building block obtained from Figure 1. For example, DWT25 system indicates a wild-type globulomer constructed by dimer building blocks, each dimer initially rotating ∼25° along the β-strand axis.

### Conformation search for monomer-based and dimer-based Aβ globulomers

Single Aβ_17–42_ peptide has a short hydrophobic N-terminal (central hydrophobic cluster (CHC) residues L_17_VFFA_21_) with two negatively charged residues (E_22_ and D_23_) and a long hydrophobic C-terminal β-strand. For each category of Aβ globulomers assembled by monomer or dimer building blocks, 72 candidates were generated, optimized, and compared in energy to determine the most likely globulomer conformations. Figure 1 shows a packing energy landscape including bonded, nonbonded, and solvation energies, calculated by the GBSW method [35], as a function of peptide self-rotation along the *z*-axis of each building block. Overall, more monomer-based Aβ globulomers suffer from large unfavorable interactions than dimer-based Aβ globulomers and the energy landscape of monomer-based Aβ globulomers is more rugged than that of dimer-based Aβ globulomers (Figure 1). This fact suggests that (i) monomer-based Aβ globulomers have less populated structures than dimer-based globulomers and (ii) the assembly of Aβ globulomers by dimers rather than monomers can greatly optimize sidechain contacts by minimizing steric confliction and electrostatic repulsion between adjacent peptides, leading to more energetically favorable conformations.

Considering the complex kinetics of Aβ aggregation, structural possibilities to assemble 12 Aβ monomers into a globulomer are very large. But, given the number of Aβ monomers, globulomers size, and possible building blocks, the search of ensemble space for the most likely globulomers could be greatly reduced. Here six representative globulomers, three from monomer-based globulomers (MWT0, MWT90, and MWT170) and the other three from dimer-based globulomers (DWT25, DWT265, and DWT330), are selected to represent typical but distinct Aβ assemblies from the lowest-energy regions (Figure 1). Overall, all initial globulomer models display a circular shape with a hollow core. Three globulomers of MWT0, DWT25, and DWT330 have N-terminal charged β-strands exposed to the bulk solution, while C-terminal β-strands buried inside to form a hydrophobic core (Figure 1). DWT330 has all adjacent β-strands entangled together via the U-turn region, causing a minor disruption of Asp23-Lys28 intrapeptide salt-bridges near the turn region, while MWT0 and DWT25 globulomers separate adjacent building blocks from each other by 2–3 Å with intact Asp23-Lys28 salt-bridges. MWT90 and DWT265 have both N-terminal and C-terminal β-strands exposed to the bulk solution with adjacent peptides entangled together. In MWT90, peptide entanglement pushes Lys_28_ residues oriented to hollow core, but negatively charged Glu_22_ and Asp23 faced to bulk solution. In DWT265, Lys28 residues form interpeptide salt-bridges with Glu_22_ and Asp_23_ of adjacent peptides, while still remain intrapeptide salt-bridges with Asp23. MWT170 is an untangle model, with an inner core composed of polar/charged N-terminal residues and an outer surface composed of hydrophobic C-terminal residues interacting with bulk water. Noted that these low-energy models prescreened by the implicit-solvent GBSW method do not necessarily imply the most stable structures in water due to the lack of explicit peptide-water interactions, thus all these preselected models are then submitted to explicit-solvent MD simulations to explore the vast variability of the conformational ensemble.

### Aβ globulomers display different structure stabilities and organizations

**Figure 2:**
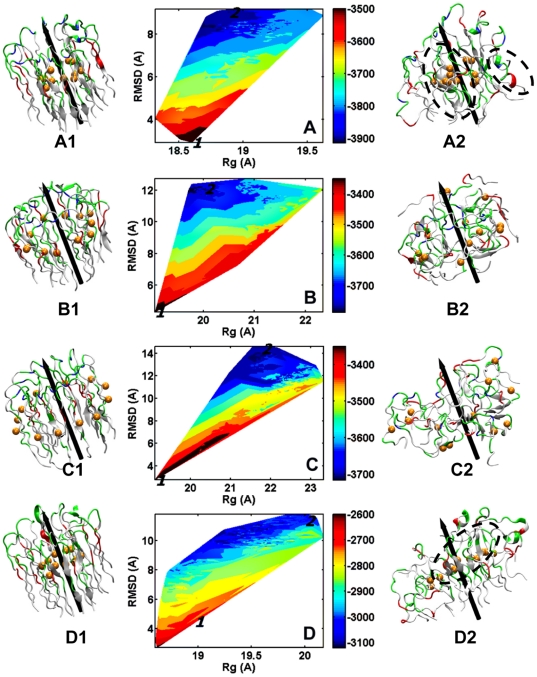
Conformational energy landscapes with respect to backbone RMSD and Rg for (A) MWT0, (B) MWT90, (C) MWT170, and (D) MMT0. Labels of 1 and 2 in the landscapes represent the initial (left) and the final (right) structures at 0 ns and 40 ns, respectively. Color codes: negatively charged residues (*red*), positively charged residues (*blue*), hydrophilic residues (*green*), and hydrophobic residues (*white*). C_β_ atoms of Met35 are shown by VDW spheres to guide eyes. All cartoon structures are rendered by VMD [62].

**Figure 3:**
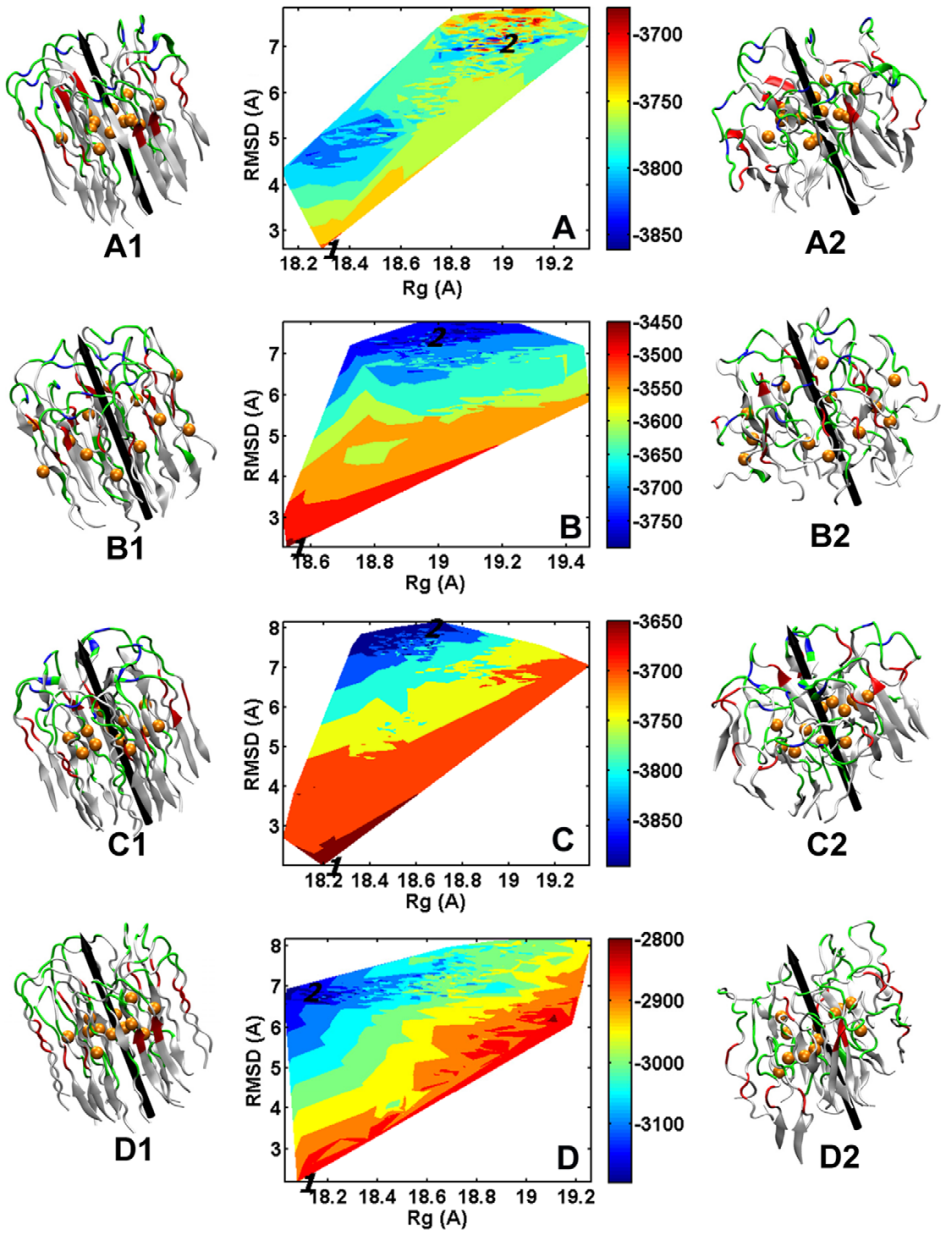
Conformational energy landscapes with respect to backbone RMSD and Rg for (A) DWT25, (B) DWT265, (C) DWT330, and (D) DMT25. Labels of 1 and 2 in the maps represent the initial (left) and the final (right) structures at 0 ns and 40 ns, respectively. Color codes: negatively charged residues (*red*), positively charged residues (*blue*), hydrophilic residues (*green*), and hydrophobic residues (*white*). C_β_ atoms of Met35 are shown by VDW spheres to guide eyes.

Figures 2 and 3 shows conformational energy landscapes of monomer-based and dimer-based globulomers as a function of root-mean-square derivation (RMSD) and radius of gyration (Rg). Initial and final states are marked as 1 and 2 on the energy landscapes and the corresponding atomic structures at 0 (left) and 40 (right) ns are shown, respectively. The energy landscapes of all globulomers show some interesting features. First, as the simulations proceed, all Aβ globulomers directly move from a narrow high-energy area to a wide low-energy basin without crossing any large energy barrier. Second, Aβ conformations are highly sampled. In the starting conformations, all Aβ globulomers have similar size and shape, with the outer diameter of ∼40 Å. During the simulations, all globulomers start to relax and swell, resulting in disordered structures by gradually losing their initial intact circular shape, as indicated by increased (RMSD, Rg) values of (7.7 Å, 18.8 Å) for MWT0, (12.0 Å, 19.9 Å) for MWT90, (14.3 Å, 21.8 Å) for MWT170, (7.0 Å, 18.8 Å) for DWT25, (7.5 Å, 19.1 Å) for DWT265, and (7.1 Å, 18.6 Å) for DWT330. On the other hand, there also exist several discrete basins with small but non-negligible structural populations, suggesting different polymorphic structures.

For monomer-based Aβ globulomers, visual inspection of MD trajectories shows that in MWT0, initial intact circular organization converts into several dynamic subunits, but these subunits still favor to associate together and form a central core by hydrophobic C-terminal β-strands whose conformations are well reversed, especially for the formation of Met35 cluster (Figure 2-a2). N-terminal β-strands that are exposed to the solvent become more disordered with large populations of random coils and disrupted intra salt bridges of Asp23-Lys28. In the initial structure of MWT90, Aβ peptides are physically entangled together via the U-turn region, with Lys28 side chains orienting to the central core. During MD simulations, repulsive electrostatic interactions induced by adjacent Lys28 residues and unfavorable interactions of exposed hydrophobic residues from both N- and C-terminal β-strands with water molecules tend to push all adjacent peptides away from each other. On the other hand, physical entanglement between adjacent peptides restrains peptide movements. The competition between these two opposite forces distorts overall peptide organization by dragging the whole structure towards different directions. In MWT170, it also suffers from large structural deviation, primarily because unfavorable hydrophobic-water interactions between the exposed hydrophobic C-terminal residues and the bulk water tend to unfold the whole structure. Meanwhile, most of intact β-structures at the N- and C-terminals are quickly lost and converted into random coils, and some intra-peptide Asp23-Lys28 are also disrupted leading to more flexible U-turn region. The loss of secondary structure and inter/intrapeptide contacts causes the whole structure to expand by ∼3 Å.

Similar to monomer-based globulomers, after MD relaxation, all dimer-based globulomers undergo some structural rearrangement by losing their initial 6-fold symmetry. In both DWT25 (untangle model) and DWT330 (entangle model) globulomers, analysis of MD trajectories shows that overall size remains roughly constant with subtle increment in radius by ∼0.5 Å for DWT25 and 0.4 Å for DWT330, and overall sphericity is slightly decreased from 0.85 to 0.78 for DWT25 and from 0.85 to 0.80 for DWT330. In contrast, DWT265 globulomer experiences large structural deviation as indicated by a continuously increased RMSD of >7.5 Å. High structural instability of the DWT265 globulomer could be attributed to its unique peptide organization, i.e. six Aβ dimers are packed into two layers of hexamers in a “shoulder-by-shoulder” way, in which the inner hexamer is entangled via turn regions and the outer hexamer is untangled and embraces the inner hexamer. Exposure of the entire outer hexamer to water leads to extremely high mobility for all external residues, and some of the peptides even diffuse from the globulomer to the solution. In addition, the disruption of intrapeptide salt bridge of Asp23-Lys28 (Table 2) by peptide entanglement at the turn region also causes a severe loss of U-bend structure. As compared to monomer-based globulomers (RMSD≈8.9–14.3 Å and Rg≈18.8–21.8 Å), dimer-based globulomers experience relative small structural deviation (RMSD≈7.1–7.4 Å and Rg≈18.6–19.1 Å), presumably due to the enhanced peptide-peptide interactions.

**Table 2:** Number of intra-peptide and inter-peptide salt bridges averaged from the last 5 ns MD simulations.

Salt bridges		MWT0	MWT90	MWT170	DWT25	DWT265	DWT330
D_23_-K_28_	Intrapeptide	4	1	1	10	6	5
	Interpeptide	8	0	7	4	4	5
E_22_-K_28_	Intrapeptide	2	0	0	4	0	2
	Interpeptide	7	1	7	2	2	3

### Heterogeneous structures suggest the polymorphilc nature of Aβ globulomers

**Figure 4:**
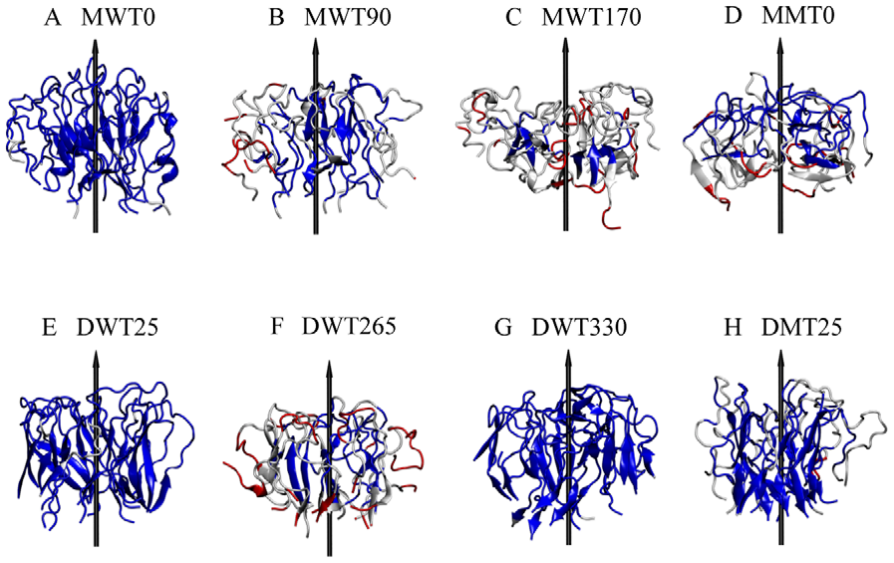
Averaged structures of monomer-based Aβ globulomers of (A) MWT0, (B) MWT90, (C) MWT170, and (D) MMT0 and dimer-based Aβ globulomers of (E) DWT25, (F) DWT265, (G) DWT330, and (H) DMT25 from the last 5-ns MD simulations. The residue-based RMSF is imposed on each averaged structure using a *blue-white-red* scale, with low RMSF of <3 Å (*blue*), intermediate RMSF of 3∼6 Å (*white*), and high RMSF of >6 Å (*red*).

Heterogeneous structures of Aβ globulomers, averaged from the last 5 ns, are presented in Figure 4. The residue-based root-mean-square fluctuations (RMSFs) are projected on the average globulomer structures to reflect residue motion, with a blue-white-red color scale (i.e. 0–3 Å for blue, 3–6 Å for white, and >6 Å for red). It can be seen that MWT90, MWT170, and DWT265 displays much larger atomic motion than MWT0, DWT25, and DWT330. Almost all residues in MWT0, DWT25, and DWT330 globulomers display very small motion of <3 Å, resulting in rigid and compact structures. Despite of structural heterogeneousness, these compact and rigid structures still share similar peptide organization where Aβ peptides associate together via their hydrophobic C-terminal parts forming a less solvent-exposed core, further suggesting that the central core is more geometrically favorable for hydrophobic interactions. Conversely, in the MWT90 and MWT170, apart from those solvent-exposed residues, large residue fluctuation is observed in turn regions in MWT90 and N-terminal β-strands in MWT170. In DWT265, due to the shoulder-by-shoulder organization, the inner peptides, which interact with each other largely through weak hydrophobic interactions, can not form a compact core, while the outer hexamer is completely exposed to solvent, once the simulations proceed, the peptides in the outer layer are ejected, causing a more loose and dynamic structures. In three cases of the MWT90, MWT170, and DWT265 globulomers, although high atomic motions are observed for most of the residues, Aβ peptides are still partially associated together.

**Figure 5:**
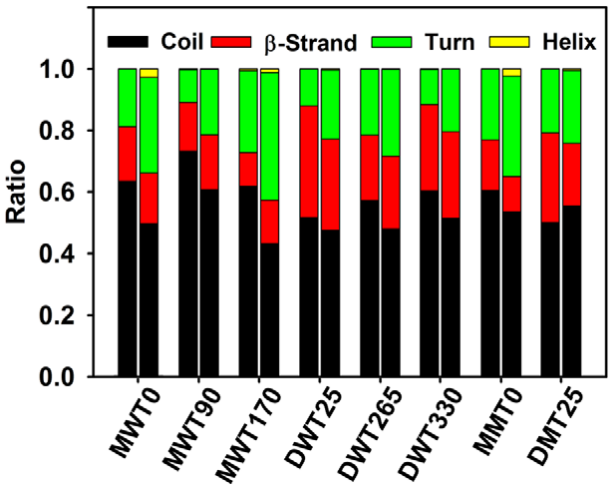
Comparison of secondary structure populations between the initial 5 ns (*left column*) and final 5 ns (*right column*) conformations for six wild-types and two mutants of Aβ globulomers.

Atomic motion changes not only overall structural organization, but also local secondary structure. Figure 5 shows the comparison of secondary structure distributions between globulomer conformations at initial and final 5 ns. As compared to over 70% β-structure in mature fibrils, all monomer-based globulomers only contain a very small amount of β-structures of ∼15–18%, with continuous decrement by ∼5% as simulations proceed. In dimer-based globulomers, coil structures are still dominant conformation, but β-strand structures are maintained at 24.4%, 18.3%, and 28.1% with <1% variations for DWT25, DWT265, and DWT330, respectively. It is well known that the cross-β structure is a structural characteristic in Aβ fibrils, protofibrils, or other high-order oligomers, but our simulations show that the globulomers exhibit heterogeneous and dynamic conformations in the presence of partially folded coils (46.7–67.6%), turns (19.8–31.1%), and β-strands (10.8–28.1%), suggesting that it is not necessary for globulomers to largely adopt well-defined β-structure especially at the very early stage of Aβ aggregation. Despite of the less populated β-structures, as a general feature, both interpeptide and intrapeptide salt bridges of Asp23-Lys28 and Glu22-Lys28 near the turn region are still survived during the simulations (Table 2), especially for stable MWT0, DWT25, and DWT330 globulomers. The maintenance of Asp23-Lys28 salt bridges helps to stabilize an intrapeptide U-shaped conformation and interpeptide association [38].

**Figure 6:**
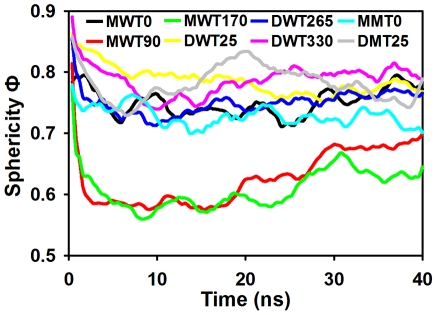
Time evolution of globulomer sphericity for DWT25 (*black*), DWT265 (*red*), DWT330 (*green*), MWT0 (*yellow*), MWT90 (*blue*), MWT170 (*pink*), D25M (*cyan*), and M0M (*gray*).

It is also interesting to quantitatively monitor the shape change of globulomers over time. The 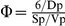 is used to measure the extent of the circular shape of an objective where D_p_ is the averaged diameter of globulomer, S_p_ is the surface area of globulomer, and V_p_ is the volume of the globulomer. The sphericity (Φ) ranges between 0 and 1 and Φ = 1.0 represents a perfect sphere. It can be seen in Figure 6 that during MD simulations, all globulomers lose their initial perfect circular shape to some extent. Monomer-based globulomers experience relatively large Φ changes from 0.78 to 0.77 for MWT0, from 0.81 to 0.70 for MWT90, and from 0.78 to 0.64 for MWT170, while dimer-based globulomers display relatively small Φ changes from 0.86 to 0.77 for DWT25, from 0.86 to 0.77 for DWT265, and from 0.89 to 0.79 for DWT330. Overall size and shape changes indicate the rearrangement of sidechain packings and associated interaction patterns.

All aggregated Aβ globulomers display a broad range of structural variations in peptide packings, with different populations on a rugged energy landscape. Using all 6 models and 3000 conformations (500 for each model) extracted from the last 10-ns MD simulations, we estimate the overall populations for each model using in-house Monte Carlo simulations. The population results are reported in Table 1. MWT0, DWT25, and DWT330 with a hydrophobic core formed by C-terminal residues represent ∼27.5%, ∼18.5%, and ∼27.8% of globulomer ensemble, appearing to be the most likely conformations. MWT90 and DWT265 occupy ∼11.7% and 11.2% of globulomer populations, suggesting minor but alternative polymorphic variants of Aβ globulomers. MWT170 with ∼3.3% population is disfavored, primarily due to the exposure of hydrophobic C-terminal residues in solution. It should be noted that all interaction energies are calculated on the basis of the last 10-ns MD trajectories, thus similar interaction distributions between monomer-based globulomers and dimer-based globulomers does not necessarily imply that monomer-based globulomers have comparable structural stability to dimer-based globulomers. Instead, for monomer-based globulomers final disordered structures are more likely conformations as compared to initial well-packed conformations, suggesting that disordered aggregates derived from both monomer-based globulomers and dimer-based globulomers can coexist in a rugged energy landscape with different populations. Additionally, Aβ globulomers are more likely to adopt heterogeneous structures with mixed coils, turns, and β-strands, rather than well-packed circular structures with dominant β-structures. Heterogeneities in golobulomer conformations with different sidechain packings, building blocks, and secondary structures further broaden a repertoire for polymorphic variants of Aβ aggregates, consistent with experimental data that some low-order soluble oligomers and globulomers appear to be relatively disordered [39].

### Stable Aβ globulomers are assembled by several dynamic subunits with a hydrophobic core

**Figure 7:**
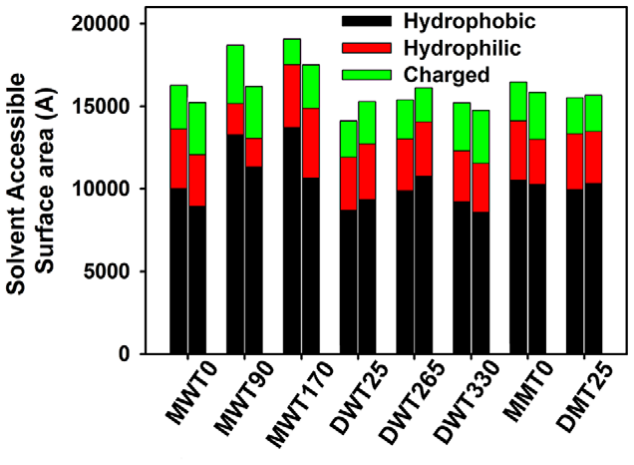
Comparison of solvent accessible surface area of hydrophobic C-terminal residues Ile31-Ala42, charged/hydrophilic N-terminal residues Leu17-Ser26, and turn residues Asn27-Ala30 between the initial 5 ns (*left column*) and the final 5 ns (*right column*) trajectories for all globulomers.

Comparison of all monomer-based and dimer-based globulomers reveal that only globulomers, whose hydrophobic C-terminals are well protected from the solvent (i.e. MWT0, DWT25, and DWT330) are able to maintain overall compact structures consistent with the NMR observation of ADDL and globulomers that highly hydrophobic C-terminal residues are largely solvent inaccessible. In both DWT25 (untangle model) and DWT330 (entangle model) globulomers, large hydrophobic patches of Met35, Val36 and Val39 at the C-terminal β-strands are observed in the center core, providing strong hydrophobic forces to associate all peptides. Similarly, in MWT0 four hydrophobic residues of Ala30, Met35, Val39 and Val40 form a cluster in the central core. To compare with H/D exchange NMR spectroscopy, we calculate and compare the solvent accessible surface area (SASA) of C-terminal (residues 31–42), N-terminal (residues 17–26), and turn regions (residues 27–30) for each model between the first 5 ns and the last 5 ns (Figure 7). It can be seen that for all Aβ globulomers hydrophilic/charged N-terminals have much large SASA values than hydrophobic C-terminals, indicating that N-terminal residues are largely exposed to the solvent. Moreover, as compared to relatively loose and dynamic globulomers of MWT90, MWT170, and DWT265, compact globulomers of MWT0, DWT25, and DWT330 have relative small SASA values for hydrophobic C-terminal residues because more C-terminal residues are buried in the core and thus protected from solvent. The decreased hydrophobic SASA accompanied with the globulomer swelling suggests that more hydrophobic residues are oriented toward the center of the globulomers to prevent from solvent access, while maintaining overall structures highly hydrated. Simulation results are consistent with amide solvent protection analysis that most backbone hydrogens of residues 30–42 near the C-terminal are inaccessible to the solvent [28], [40]. Both computational and experimental results suggest that strong hydrophobic interactions contributed by C-terminal residues in the core play an important role in the formation of Aβ globulomers. On the other hand, these aggregated globulomers contain several dynamic subunits with large portion of disordered β-strands. Since ADDL and globulomers are often observed at the very early stage of Aβ aggregation, it probably is not necessary for Aβ peptides to adopt a perfect spherical/annular morphology in globulomers with a large portion of β-structure. The aggregated globulomer conformations are very similar to disordered annular structures observed in lipid bilayers by AFM and MD simulations [41], [42] showing several discrete subunits surrounding a central axis.

### Solvation and VDW interactions control the structure and dynamics of Aβ globulomers

**Figure 8:**
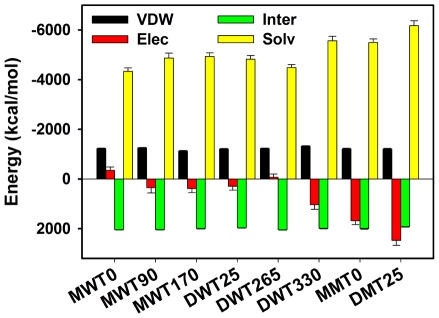
Energy decomposition for all globulomers averaged from the last 10 ns simulations.

Comparison among various Aβ globulomers reveals different dynamical behaviors, strongly depending on Aβ packings such as packing distance and orientation between peptides, sidechain contact pattern at the juxtaposed interface, and possibly conformational changes of peptides. All these factors can be directly lumped and assessed by peptide-peptide and peptide-water interactions. To further understand physical driving forces stabilizing Aβ globulomers, interaction energies of various Aβ globulomers including VDW, electrostatic, bonded, and solvation interactions are averaged and compared from the last 10-ns using GBSW method (Figure 8). Surprisingly, all six wide-type globulomers display similar interaction distributions, although they possess significant diversity in structural organization and dynamics. Energy decomposition shows that the solvation energy (from −4356 to −6173 kcal/mol) plays a pronounced role in stabilizing the globulomers. Similar to native protein folding, most of amyloidogenic peptides (except GNNQQNY) require some hydrophobic interactions to drive peptide aggregation and form a dehydrated or less hydrated interior core by expulsing waters from peptides. Expulsion of waters from peptides is the first and obligatory step to facilitate initial peptide association and subsequent fibrillization by reducing free energy barriers arising from dehydration entropic effects [43], [44], [45]. Once the stable aggregates form, highly hydrated water layer around the aggregate surface in turn helps to prevent peptide disassociation. Clearly, the VDW interactions ranging from −1206 to −1324 kcal/mol play a dominant role in peptide-peptide interactions, contributing all favorable interactions to peptide association. The electrostatic energy contributes minor or even unfavorable interactions to globulomer structures. Large unfavorable electrostatic interactions in DWT330 arise from the intertwinement of negatively charged residues of Glu22 and Asp23 near the turn region. Internal energies (1921∼2046 kcal/mol) present stable energetic contributions to maintain secondary structures of Aβ globulomers.

**Figure 9:**
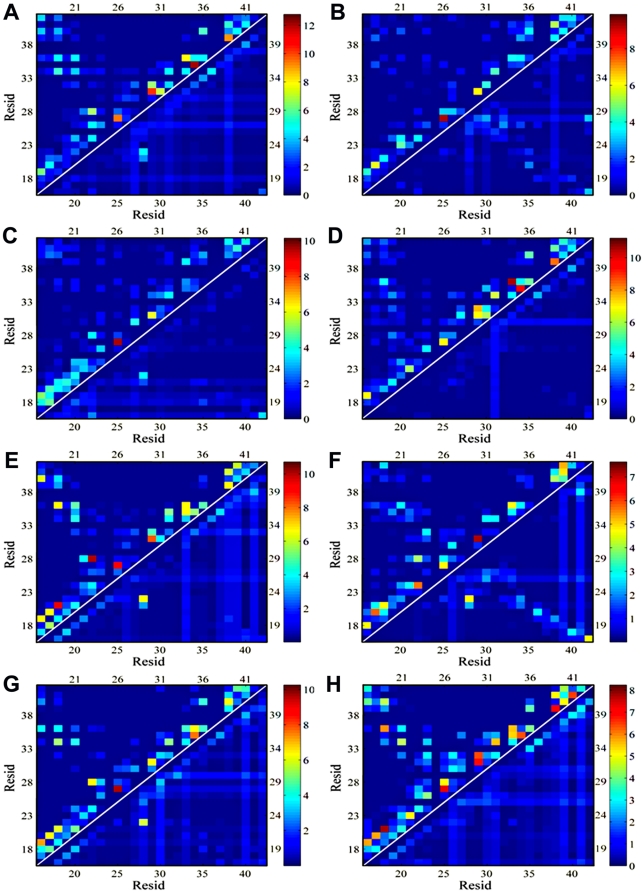
Residue-residue interaction maps including sidechain contacts (*upper left triangular corner*) and hydrogen bonds (*lower right triangular corner*) for (A) MWT0, (B) MWT90, (C) MWT170, (D) MMT0, (E) DWT25, (F) DWT265, (G) DWT330, and (H) DMT25. A sidechain contact is defined if the center of mass of sidechains between two residues is less than 6.0 Å. A hydrogen bond is defined if donor-acceptor distance is <3.5 Å and accepter-donor-hydrogen angle is >120°.

To further reveal the correlation between overall structural conformation and underlying residue-based interactions, Figure 9 shows residue-based sidechain contacts (upper triangle) and hydrogen bonds (lower triangle) between peptides for all globulomers. It can be seen that both sidechain contacts and hydrogen bonds are mainly derived from interactions between adjacent residues (i.e. peptide i and peptide i+1), and sidechain contacts appear to be dominant forces in peptide association, as compared to hydrogen bonds. It is conceivable that at the early aggregation stage, the rapid formation of a disordered assembly is mainly driven by nonspecific sidechain contacts especially by strong hydrophobic interactions, while subsequent slow structural reorganization from the disordered aggregates to ordered β-structure-rich aggregates is directed by specific interchain hydrogen bonds [46]. For MWT0, DWT25, and DWT330, besides adjacent residue interactions (along the right diagonal), there is additional intensive contact region between L_17_VPPA_21_ from N-terminals and L_34_MVGGVVIA_42_ from C-terminals of Aβ globulomers, suggesting that hydrophobic interactions in these regions additionally provide strong forces to associate peptides. Especially, six core-forming residues of Phe19, Ala21, Leu34, Met35, Val36, and Val39 provide sufficient hydrophobic interactions to the overall stability of globulomers. In comparison, inter-peptide interactions of Phe19 with Leu34, Ala21 with Leu34, Phe19 with Val36, and Ala21 with Val36 are either eliminated or greatly reduced in other globulomer models.

### Dimer is a basic building block for Aβ globulomers

**Figure 10:**
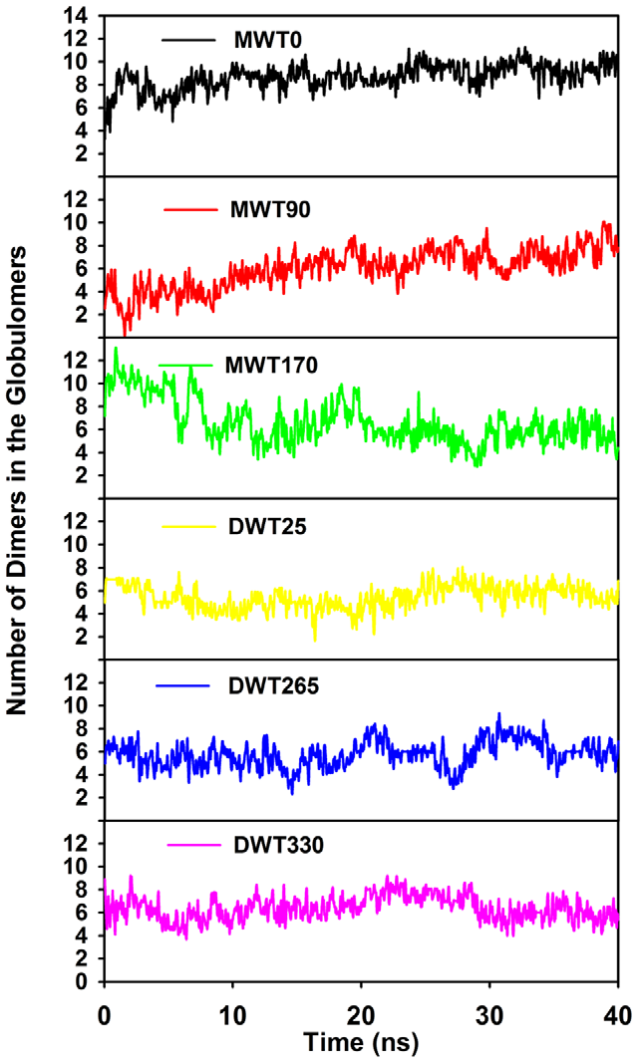
Number of dimers formed in the Aβ globulomers during 40 ns MD simulations.

Even for the most stable monomer-based model of MWT0, a close inspection of MD trajectories reveals that all monomers tend to dimerize into different subunits. It has been hypothesized that Aβ dimers may be the basic and smallest build blocks to form high-ordered Aβ oligomers [47], [48]. Based on our MD results and experimental data, Aβ dimerization is expected to be favored by hydrophobic interactions arising from the minimal sidechain contacts between the C-terminals [49]. To quantitatively characterize the dimer population in all Aβ globulomers during MD simulations, we performed additional 40-ns MD simulation of Aβ dimer (the same as dimer building block in globulomer) in bulk solution and the last 20-ns MD trajectory is used to generate a dataset containing 5000 interpeptide sidechain contacts between C-terminals and 5000 interpeptide interaction energy computed at every 40 ps. Both C-terminal sidechain contacts and interpeptide interaction energies are used as dynamic references to determine whether a dimer is formed in the globulomers. Specifically, two peptides in the globulomers are sequentially selected and their C-terminal sidechain contacts and interpeptide interaction are computed and compared to 3000 references randomly selected from the dataset. If over 75% of 3000 comparisons shows that either C-terminal sidechain contacts are greater than the randomly selected references or the interaction energies are more favorable than the references, these two peptides are considered to form a dimer. Figure 10 shows that although all Aβ globulomers display significant structural heterogeneousness with different peptide packings, they tend to be dimerized in order to maintain global structures. Even for the most unlikely MWT170 model (only ∼3.3% population), it still can achieve ∼4 dimers, while all other five globulomers are able to maintain ∼6 pairs of dimer. We also observe that two dimers can form a tetramer in the MWT0 model. This fact suggests that the formation of dimer building blocks is critical for maintaining globulomer's stability.

**Figure 11:**
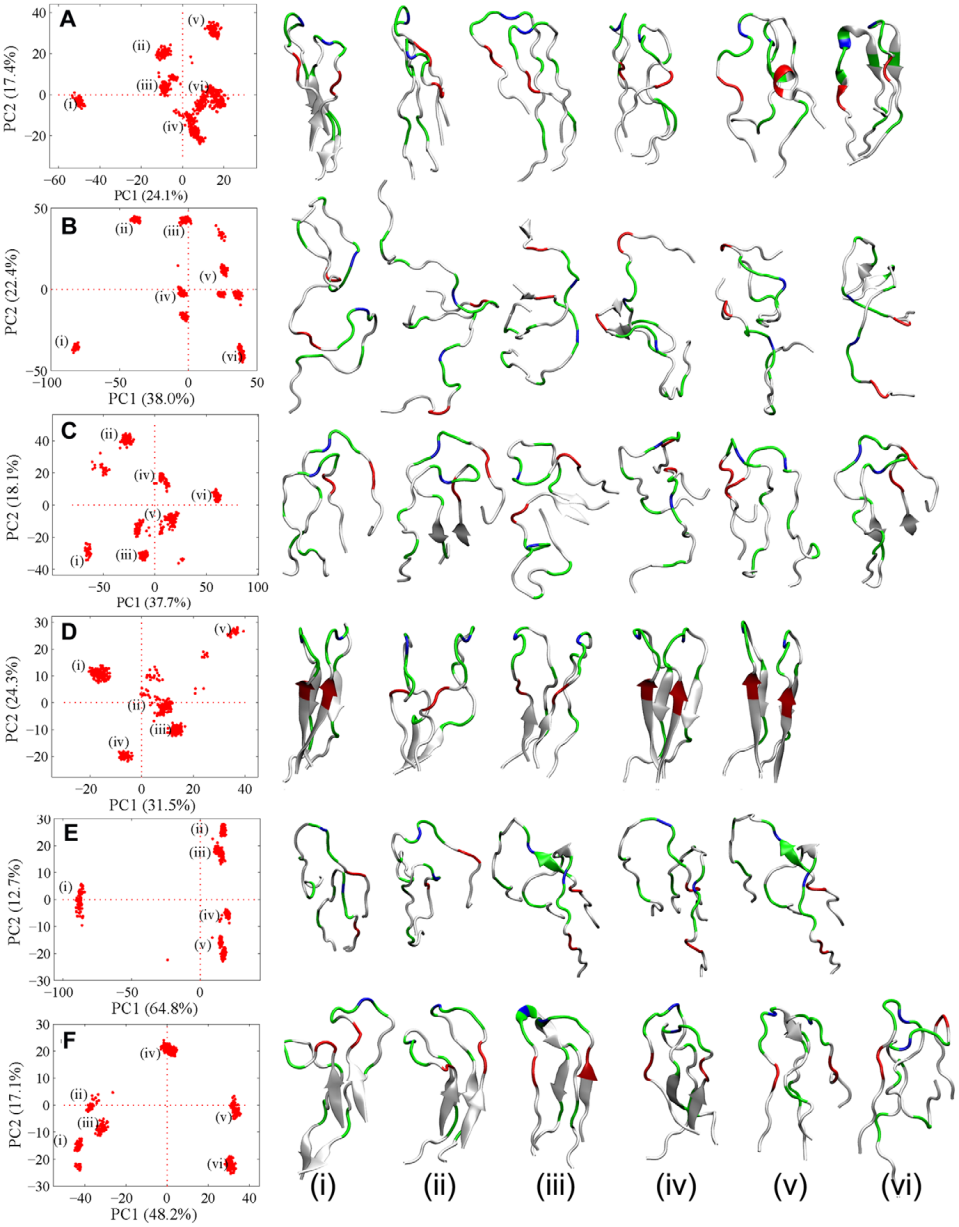
The most populated Aβ dimeric clusters extracted from the condensed areas of principle component analysis maps for (A) MWT0, (B) MWT90, (C) MWT170, (D) DWT25, (E) DWT265, and (F) DWT330.

To study the conformation distribution of the dimers, the 1000 configurations of each globulomeric model are clustered into 5 or 6 representative dimer groups based on the principle component analysis (PCA). A diversity of dimeric structures extracted from the predominant clustered area in the PCA maps is shown in Figure 11. It can be clearly seen that most of individual peptides from the compact models of MWT0, DWT25, and DWT330 remain U-bend conformation, but some peptides from the loosely packed models of MWT90, MWT170, and DWT265 partially adopt disordered conformation. In the compact models, individual peptides are likely to pack together with an approximate parallel alignment with respect to each other (Figure 11A, D, F). In these dimers intermolecular hydrogen bonds are formed between both C- and N-terminal residues. In contrast to largely registered dimers with similar parallel β-sheet conformation, a wide variety of disordered dimers are scattered in the PCA maps, with a complete out-of-register alignment of the peptides due to the loss of specific interpeptide hydrogen bonds. Consistently, dimeric structures in the compact MWT0, DWT25, and DWT330 models have much smaller SASA values than those structures in the MWT90, MWT170, and DWT265 models, suggesting that Aβ dimerization is more favored by the partially desolvation of the peptides. By correlating dimer clusters with interaction energies, it appears that the compact dimer conformation with an approximate parallel alignment is presumably more favored than the out-of-register dimer conformation in the globulomers.

### Lys28Ala mutation suggests the importance of dimerization on the stability of Aβ globulomers

It is well known that Asp23-Lys28 and Glu22-Lys28 salt-bridges play an important role in Aβ folding and aggregation by facilitating and stabilizing the formation of β-hairpin structure [20], [50]. To computationally examine the effect of these salt bridges on the overall structural organization of Aβ globulomers, we perform two additional MD simulations by substituting Lys28 with Ala residues in the stable MWT0 and DWT25 globulomers. Visual inspection of MD trajectories also shows that monomer-based mutant of MMT0 quickly loses their initial circular shape at 5 ns and transforms into a flat elongated shape (Figure 2-d2). The deformation is quantified by largely increased RMSD of 11.5 Å and Rg of 19.7 Å, as compared to RMSD of 8.9 Å and Rg of 18.8 Å in wild type. In contrast, dimer-based mutant of DMT25 remains similar size and shape to the wild type, with comparable RMSD (7.5 Å for mutant vs. 7.0 Å for wild type), Rg (18.8 Å vs. 18.8 Å), and Φ (0.77 vs. 0.78). Consistently, Figure 4 shows that mutant MMT0 is more flexible than mutant DMT25.

Since both mutants have similar initial peptide organization: (i) C-terminal residues form a hydrophobic core while N-terminal residues expose to solvent; (ii) no peptide entanglement is involved; and (iii) initial Asp23-Lys28 and Glu22-Lys28 salt bridges are intact and locate at similar position, the different effects of eliminating salt bridges on the structural stability of two mutants can be largely attributed to the difference in building blocks. Our results show that the Lys28Ala mutations significantly alter electrostatic interactions, not other interaction energies for both mutants. Specifically, the neutralization of Lys28 by Ala eliminates favorable electrostatic interactions of Asp23-Lys28 and Glu22-Lys28, leading to large repulsive unfavorable electrostatic energies increased from −342.6 kcal/mol to 1683.4 kcal/mol for MMT0 and from 295.8 kcal/mol to 2474.2 kcal/mol for DMT25 (Figure 8). Meanwhile, it can be seen in Figure 9 that the Lys28Ala mutations greatly enhance residue-residue contacts among Val39, Val40, and Ile41 for DMT25, but not for MMT0, which could be simply attributed to Aβ building blocks. The peptide-peptide distances in DMT25 globulomer composed of Aβ dimers are relative smaller than those in MMT0 globulomer composed of Aβ monomers. Consistently, due to relative small separation distance between peptides in DMT25 globulomer, DMT25 globulomer suffers from larger unfavorable electrostatic repulsion than MMT0 globulomer. Thus, competition between the loss of favorable electrostatic interactions and the gain of favorable hydrophobic interactions leads to different final structures. Taken together, it appears that the formation of dimer is a key step for maintaining overall globulomer structures, consistent with our computational and experimental observations [28].

## Discussion

Similar to other amyloidgenic peptides, Aβ peptides are highly polymorphic and can aggregate into different soluble oligomers with distinct structural morphology. These soluble oligomers including ADDL and globulomers have been found to be highly toxic to neurons. But, considerably less is known about the molecular structure of Aβ oligomers and their relation to amyloid fibril formation and amyloid neurotoxicity. Our computational results provide insights into structural characterization of Aβ globulomers, illustrating several interesting observations in globular structures. It is of paramount importance to compare our simulation models to experimental data, although they are probing structures and dynamics at different length scales and time scales. First, stable Aβ globulomers (i.e. MWT0, DWT25, and DWT330) adopt different conformations assembled by several dynamic dimer subunits, but they also share a common structural characteristic that the C-terminal residues form an interior hydrophobic core of a globular structure, while the hydrophilic/charged N-terminal residues are largely exposed to the solvent to make the whole structure soluble. These structural information from computational models is in good agreement with amide protection analysis from H/D exchange NMR spectroscopy, showing that amides of residues Ile31-Ile40 at the C-terminals are fully protected and exhibit very slow amide exchange [28]. Ma and Nussinov [51] recently proposed three computational models of Aβ globulomers. Although these three globulomer models adopted different turn conformations (the Luhrs turn [20] vs. the Tycko turn [52]) and β-strand association (parallel vs. antiparallel), they indeed shared a common structural characteristics that both hydrophobic C-terminal β-sheets from two Aβ hexamers were orthogonally intertwined together and embedded into the interior core of β-strand-turn-β-strand region, while hydrophilic/charged N-terminal β-sheets were exposed to solvent. This intertwined conformation prevents not only the disassociation of single peptide from the globulomer, but also the association of other oligomers with the globulomer. Our models are significantly different from their model in peptide organization, but Ma-Nussinov and our models emphasize the importance of strong C-terminal interactions and satisfy experimental observations in size, molecular weight, and H/D amide solvation protection, suggesting that Aβ globulomers could adopt different polymorphic conformations. Additionally, a number of experimental studies of other Aβ oligomers including 2-fold double-layer 12-mer, 3-fold triangular 18-mer, and 5-fold disk-like pentamers [18], [53], [54] have also shown that C-terminal residues are well protected from solvent exchange, which appears to be a signature structure for Aβ oligomer/globulomer. It is conceivable that highly charged and hydrophilic N-terminals in the surface layer, especially for residues 1–16, can facilitate specific interactions with negatively charged lipid bilayer and cell membrane via electrostatic interactions, leading to membrane penetration/disruption and subsequent unregulated ion leakage and ultimately cell death. Thus, interruption of C-terminal residues aggregation to form a hydrophobic core may provide a potential route to inhibit or reduce Aβ toxicity.

Second, all Aβ globulomers lose their initial structural symmetry and perfect annular shape, but some of them still remain a compact structure with dominant coils (∼60%) and non-negligible β-structures (∼20%). Although there is no secondary structure analysis available for Aβ globulomers, a number experimental studies have shown that some low-order soluble oligomers seem to be relatively disordered with mixed secondary structures [39], [55]. Ahmed and co-workers [54] recently reported that no defined β-sheet structure was observed until Aβ42 oligomers covert into protofibrils and fibrils. As compared to dominant cross-β-sheet in mature fibrils, *in vitro* globulomers appear to have amorphous structures with mixed peptide conformations. The major differences between Aβ fibrils and globulomers center on their structures and kinetic pathways. In fibrils, the formation of fibrils *in vitro* is a two-phase procedure via a lag phase by nucleation seed formation, followed by a growth phase by association of monomers to protofibrils, and usually takes sufficient time (i.e. days or weeks) for peptides to undergo structural transition and aggregation depending on experimental conditions. Residues in the fibrils are highly registered within the sheets and highly complementary between the sheets, with dominant cross-β-structures [21], [56], [57]. Barghorn et al. [11], [15] found that Aβ-globulomer is independent from the pathway of fibril formation and inert to transform into Aβ fibrils by interacting with other Aβ monomers and oligomers. Our simulation models confirm that once Aβ globulomer is formed and stabilized, circular curvature (sphericity ∼0.78) and compact structure of Aβ globulomers, especially involving peptide entanglements in our DWT330 model and Ma's model, present a good shield to prevent fibril elongation and lateral association from adding Aβ peptides into existing globulomers, suggesting that Aβ globulomers are off-pathway species to fibrils. Bellesia and Shea [58] also showed that as the peptide β-sheet propensity decreases, peptides are more likely to be form off-pathway aggregates which do not evolve into fibrils. Additionally, since the formation of Aβ globulomer is a rather quick procedure and usually occurs at the very early stage of aggregation, residues are largely packed in an out-of-register manner between the twisted β-strands, reflecting large population shifts in different polymorphic conformations. Our stable globulomers have overall size of ∼40 Å, in a good agreement with AFM imaging sizes of 40–50 Å and molecular weight of ∼64 kDa corresponding to 12–16 peptides in Aβ globulomers [28].

Finally, although Aβ globulomers exhibit heterogeneous shapes and structures in a rugged energy landscape, they are more preferred to be organized by dimer or tetramers through dimer-dimer association. Even for monomer-based globulomers, for example, final structure of MWT0 model is reorganized into two tetramers and two dimers. Dimer and tetramer are typically species and often observed in *in vitro* experiments [59]. Lys28Ala mutations lead to inconsistent structural stability on monomer-based MWT0 and dimer-based DWT25, further confirming that dimers rather than monomers are more energetically favorable building blocks for Aβ aggregation. But, structural comparison of simulated Aβ dimer with experimental derived dimer in globulomers reveals certain discrepancies in overall peptide packing organizations and local secondary structures. In our Aβ globulomers, two twisted peptides adopt an interpeptide parallel β-sheet associated by two antiparallel U-bend monomers consisting of two antiparallel β-strands (residues 17–25 and 31–42) linked by a U-turn region (Ser26-Asn27-Lys28-Gly29-Ala30). In experimentally proposed dimer structure in Aβ globulomers [28], monomeric Aβ consists of three β-strands (β1: residues 17–23, β2: residues 28–33, and β3: 34–42) and one bend (residues 24–27). β1 and β2 form an intrapeptide antiparallel β-sheet, which is separated from another intrapeptide β-sheet of the mating peptide. Two β3 from each peptide form an interpeptide parallel β-sheet, which provides sufficient hydrophobic forces to associate two peptides together. The whole dimer structure displays a Y shape having both intermolecular and intramolecular β-sheet contacts. The discrepancy in the dimer conformation between experiments and simulations is likely attributed to elevated Aβ concentration and different solvent used in our simulations. Aβ concentration in the simulations is in the range of millimolar, which is much larger than micromolar concentration *in vitro* experiments. Yu et al. [28] prepared Aβ globulomers in the presence of very dilute hydrocarbon detergent of 0.05%, which can facilitate Aβ_1–42_ peptides to convert into globulomers. Small changes in the experimental conditions can easily shift the population towards different structures via different pathways. Very few papers [60], [61] have used full-length Aβ_1–42_ peptide to study the structure and dynamics of Aβ oligomers and fibrils, presumably because N-terminal residues of 1–16 are highly disordered and not atomic structure is available to date. Thus, our Aβ globulomer models did not include N-terminal residues of 1–16. On the other hand, even if the residues 1–16 are presented in our globulomer models, these residues should have little effect on the hydrophobic core and annular packing in those stable globulomers (DWT25, DWT330 and MWT0) whose N-terminals are completely exposed to solvent.

### Conclusions

Two types of Aβ globulomers with different structural symmetries are computationally modeled by parallel alignment of 12 Aβ monomers or 6 Aβ dimers into annular structures, followed by explicit-solvent MD simulations to assess the structure, dynamics, and population of these Aβ globulomers. Regardless of initial conformations, Aβ globulomers tend to develop into different polymorphic structures with different populations. Stable Aβ globulomers are mainly composed of several dynamic dimeric subunits by packing hydrophobic C-terminals inside to form a hydrophobic core and exposing hydrophilic and negatively charged N-terminals outside to make overall structure soluble and to promote membrane interactions. Our globulomer models are in agreement with experimental observations in size, subunit organization, and molecular weight from SDS-PAGE, AFM images, H/D exchange solvent protection, and mutation studies. Detailed structural information of Aβ globulomers highlights the importance of C-terminal strands for aggregation, which may serve as potential targets for the rational design of anti-Alzheimer drugs.

It behooves us to note that since amyloid landscape is highly polymorphic and simulations cannot afford to explore all structural possibilities, our Aβ glolubomers do not necessarily rule out other heterogeneous structures. For example, as shown in Figure 1, many local energy minimums may also present some different Aβ globulomeric structures that have not been examined in this work. Additionally, existing Aβ globulomers proposed by Ma and Nussinov [51], Yu et al. [28], and us display dramatic differences in peptide packings, although they share some common structural characteristics. Further experimental studies are thus necessary to examine these computational structures of Aβ globulomers.
